# Impact of type 1 diabetes mellitus and celiac disease on nutrition and quality of life

**DOI:** 10.1038/nutd.2016.43

**Published:** 2017-01-09

**Authors:** J G Nunes-Silva, V S Nunes, R P Schwartz, S MLSS Trecco, D Evazian, M L Correa-Giannella, M Nery, M S Queiroz

**Affiliations:** 1 Nutrition and Dietetics Division, Central Institute of Clinics Hospital, University of São Paulo Medical School, São Paulo, Brazil; 2 Lipids Laboratory (LIM-10), Endocrinology and Metabolism Division of Hospital das Clinicas, Faculty of Medical Sciences, University of Sao Paulo, São Paulo, Brazil; 3 Radiology Institute of Clinics Hospital, University of São Paulo Medical School, São Paulo, Brazil; 4 Cellular and Molecular Endocrinology Laboratory (LIM-18), University of São Paulo Medical School, São Paulo, Brazil; 5 Endocrinology Division, Internal Medicine Department, University of São Paulo Medical School, São Paulo, Brazil

## Abstract

**Objective:**

Type 1 diabetes mellitus (T1DM) and celiac disease (CD) are autoimmune diseases and have similar genetic patterns. T1DM treatment is based on diet, physical activity and insulin therapy, whereas CD depends on dietary changes with restriction of wheat, rye and barley. The aim of the study was to evaluate the quality of life (QoL) of individuals with the association of T1DM and CD, to characterize their nutritional status and to compare it with those with only one disease and healthier controls.

**Subjects/Methods:**

Sixty patients controlled by sex, age and body mass index (BMI) were stratified by previous diagnosis in: T1DM and CD (DMCD group); T1DM (DM group); CD (CD group); or healthy participants (HC). The SF-36 questionnaire was applied to assess psychological well being and results were compared with glycemic control and presence of complications related to diabetes, adhesion to gluten-free diet (GFD). Nutritional status and body mass composition were determined by BMI, waist circumference, bioimpedance, general laboratory tests and whole-body densitometry.

**Results:**

The time of diagnosis of T1DM was similar between DMCD and DM groups; however, the duration of CD was significantly higher in the CD group compared with DMCD. The SF-36 analysis revealed statistically significant differences between DM and HC groups in two domains: general health (*P*=0.042) and energy/vitality (*P*=0.012). QoL was also correlated with compliance to a GFD, and scores were similar in both groups: DMCD and CD. Forty percent of individuals in the CD group had visceral fat area above 100 cm^2^, as opposed to 20% in the other groups.

**Conclusions:**

Individuals of DMCD group had similar scores to DM, CD and HC on QoL, as well as on their nutritional status and bone metabolism. Thereby, we should conclude that the association of T1DM and CD did not deteriorate their health status.

## Introduction

Type 1 diabetes mellitus (T1DM) and celiac disease (CD) are autoimmune diseases caused by the interactions of genetic and environmental factors. Both diseases have genetic patterns linked to HLA-DQ2 and HLA-DQ8, resulting in a reported prevalence of CD in T1DM five to seven times higher than in the general population.^1^ T1DM treatment is based on the regular physical activity, and nutritional and insulin therapy.^2^ Hence, the medical prescription of multiple doses of insulin and carbohydrate counting showed better glycemic control in individuals with DM1, avoiding the glucose restriction and the negative impact of dietary restriction.^3^

Similarly, treatment for CD is based on the dietary therapy, with restriction of wheat, rye and barley, responsible for the immune system activation and intestinal damage. Proper gluten restriction results in a recovery of the CD enteropathy, despite conflicting results about the adequacy of daily requirements of micronutrients and macronutrients. The appropriateness of nutrients is related to the availability, composition of gluten-free food, cultural aspects, access to ‘new diets' and specific nutritional recommendations for each population.^4, 5, 6^ Overall, individuals with adequate adhesion to the gluten-free diet (GFD) tend to consume smaller amounts of fibers, iron, calcium, folic acid and vitamin B12.^7, 8^

Some studies have shown an improvement in quality of life scores (QoL) for those patients with T1DM after initiating carbohydrate counting and intensive insulin therapy, but the need for full-time attention to the disease for a long time may deteriorate their QoL.^9, 10^ Initially, the QoL of celiac patients is affected by diet restriction; however, it improves ~1 year after the introduction of GFD, even in those with a partial adherence.^11, 12^ Despite the high prevalence of the association between T1DM and CD, the repercussion of dietary changes imposed by both diseases on QoL has been poorly evaluated. Therefore, the aim of the study was to evaluate the QoL of individuals with the association of T1DM and CD, and to characterize their nutritional status, nutritional behavior and deficiencies, comparing it with those with only one disease (T1DM or CD) and healthy controls

## Patients and methods

### Patients

To evaluate the nutritional status, and characterize usual dietary intake and impact on QoL of individuals with T1DM associated with CD, without biases related to sex, age and body mass index (BMI), we used the parameters of the 15 patients enrolled in the DMCD group to match with those of participants from the other three groups. Sixty patients controlled by sex, age and BMI were stratified by previous diagnosis in four groups: T1DM and CD (DMCD group); T1DM (DM group); CD (CD group); or healthy participants (HC group). They were recruited from Hospital das Clinicas da Faculdade de Medicina, Universidade São Paulo, endocrinology and gastroenterology outpatient clinics, although healthy participants were recruited from the nutrition and dietetics division. We adopted as inclusion criteria: (1) adult individuals aged 18–59 years; (2) type 1 diabetes diagnosed by presence of antibodies anti-glutamic acid decarboxylase or anti-islet or C-peptide <0.5 ng ml^−1^ or dependence on insulin since diagnosis of the disease; (3) CD confirmed by antibodies, anti-endomysial antibodies (anti-EMA) or anti-transglutaminase antibody positives and typical histologic alterations on intestinal biopsy. The Institutional Review Board at Hospital das Clinicas approved these studies; all participants provided signed, informed consent. All patients with diabetes were on intensive multiple doses insulin therapy, just one patient at DMCD group was with continuous subcutaneous insulin infusion. The average dose of insulin was 0.69±0.37 and 0.61±0.19 units per kg per day, group DMCD and CD, respectively.

### Nutritional status and body mass composition

The area of visceral fat was measured using octopolar bioimpedance (InBody720, Biospace Co. Ltd., South Korea), whereas total body fat and bone mineral density were assessed by whole-body densitometry (dual energy X-ray absorptiometry, Hologic Discovery W; Bedford, MA 01730-1401, USA). The registered dietitian nutritionist responsible for the project execution plan (Joyce Gouveia Nunes-Silva-S) determined nutritional status by BMI and waist circumference, assessed food intake by a 3-day food record and calculated nutritional composition using the Virtual Nutri Plus software (Copyright 2012—www.keeple.com.br) and the Brazilian Food Composition Table, TACO^13^ and Philippi.^14^ Results of macronutrients, fibers and cholesterol were compared with the recommendations of the Brazilian Society of Diabetes,^15^ and micronutrients to the references of the Institute of Medicine.^16, 17^

### Psychological well-being assessment

We applied the Brazilian version of the SF-36 questionnaire^18^ and compared data on QoL among all four groups. The value for each domain of SF-36 ranges from 0 to 100 (0 being the worst and 100, the best state of health). For groups with T1DM, SF-36 results were also associated with glycemic control and presence of complications related to diabetes, whereas for those with CD, adhesion to diet assessed by Celiac Dietary Adherence Test (CDAT)^19^ and anti-EMA were used for comparison. To infer the degree of commitment to the GFD, we used as criterion for CDAT: good-diet adhesion, scores <13, low-diet adhesion and scores >17, as suggested by the authors.^19^ A single investigator administered both questionnaires, SF-36 and CDAT during the interview for nutritional assessment.

### Laboratory methods

Anti-EMA were detected by indirect immunofluorescence against human umbilical cord, adopting <1/10 as cutoff. Glycated hemoglobin (normal range 4–6%) was determined in whole blood using ion-exchange high-performance liquid chromatography. General laboratory tests and other biochemical analyses were carried out using commercial kits, as part of the routine assessment after overnight fasting.

### Statistical analyses

We used Excel 2011 and SPSS version 20.0 (IBM SPSS Statistics for Windows, IBM Corp., Armonk, NY, USA) for statistical analysis. Variables were described as mean and s.d., median and range (minimum and maximum). The analysis of variance test was applied to analyze variance, whereas normality of data distribution was assumed within each group using the Kolmogorov–Smirnov test. For variables with statistically significant differences between groups, we performed the Bonferroni test for multiple comparisons. We applied the non-parametric test of Fisher and Pearson correlation coefficient to analyze independent variables, and to assess the relationship between the variables, respectively. Tests were performed with a significance level of 5%.

## Results

Clinical details are listed in Table 1 and are equal regarding age, sex and BMI in all groups. The duration of T1DM was similar between DMCD and DM groups; however, the duration of CD was significantly higher in the CD group compared to DMCD (*P*=0.0015).

**Figure 1:**
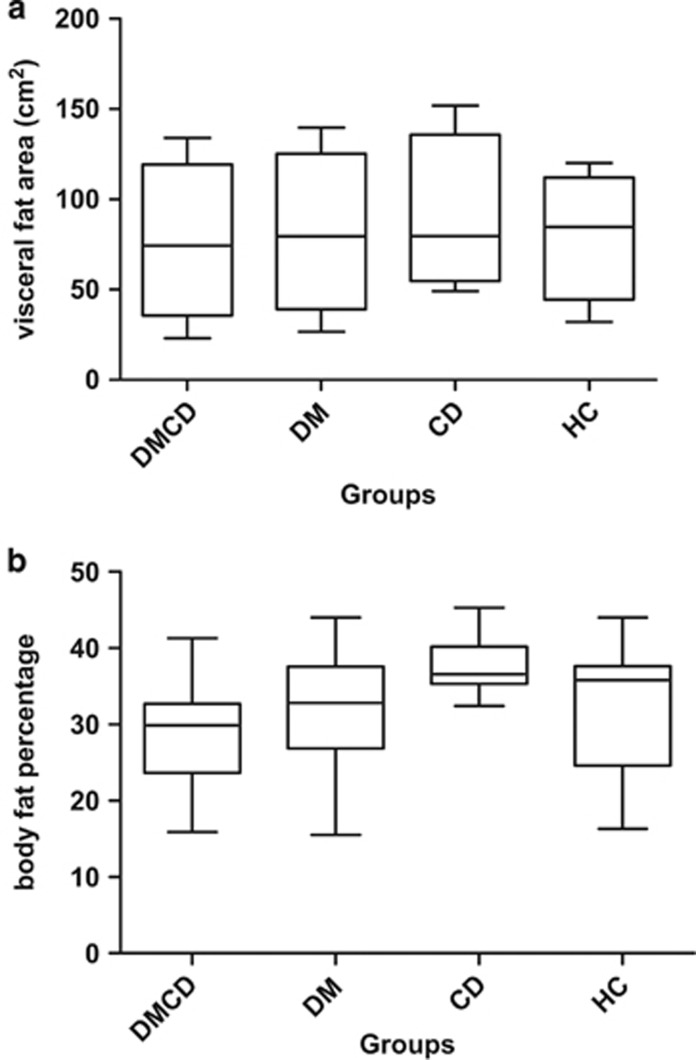
Visceral fat area and percentage of body fat according to dual energy X-ray absorptiometry ( DEXA) among all four different groups. CD, celiac disease only; DM, type 1 diabetes only; DMDC, type 1 diabetes mellitus and celiac disease; HC, healthy control. Values are expressed as means ±s.d., median and range (minimum and maximum). (**a**) Visceral fat area (cm^2^) and (**b**) body fat percentage.

As shown in Figure 1, mean visceral fat area and body fat percentages were similar among all groups. Yet, 50% of women in the CD group had visceral fat area above 100 cm^2^, whereas only 33% of patients achieved this cutoff in the other groups (Table 2). In general, 64% of women participants were considered as having increased risk for metabolic complications, according to the criteria of the World Health Organization.^20^ On the other hand, all male patients had BMI <25 kg m^−2^, waist circumference <94 cm and visceral fat <100 cm^2^. Taken together, these data pointed to similarities of clinical characteristics between groups and the tendency of women with CD to have higher risk of metabolic complications.

All participants had normocaloric habitual food intake with nutritional composition appropriately distributed by macronutrients according to standards of the Institute of Medicine^16, 17^ for the adult population. The quality of diets was adequate for carbohydrates (except for the DM group) and protein, but hyperlipidemic (except for HC). The consumption of B12 vitamin and selenium was satisfactory, whereas fiber, calcium and vitamin D intake did not reach the daily recommendations in all groups. Laboratory measurements of vitamins and minerals were similar in all groups, and within the normal range (Table 3). Nonetheless, serum levels of folic acid and magnesium were lower in individuals with CD (DMCD and CD groups) and patients with diabetes (DMCD and DM groups), respectively (Table 4). In general, it is possible to conclude that having DM1, CD or both diseases did not affect the quality of food ingested in all groups.

We also assessed bone metabolism as shown in Table 3. Our data show that all laboratorial parameters measured were similar in all groups, with only three patients with *T-*score compatible with osteopenia by densitometry: one patient of DM group and other two female participants, postmenopausal, aged 48 and 57 years old. One of these belonged to the control group and one to CD group, reflecting that almost all patients in all groups were within the normal range for *Z-*score in the bone mineral density, without any statistical difference.

Results of psychological well-being assessment estimated by the SF-36 questionnaire showed a lower score for functional capacity, physical limitations, pain and social aspects in the DM and CD groups, but without reaching statistical difference (Table 5). Two domains of the SF-36 reached statistically significant differences between DM and healthy control groups: general health (*P*=0.042) and energy/vitality (*P*=0.012). Of particular note was the negative correlation between glycemic control, assessed by glycated hemoglobin levels of the DM group, with the domain energy/vitality (Table 6).

Complications associated with T1DM were not correlated with a significant difference in health status (*P*=0.22), vitality (*P*=0.22), pain (*P*=0.27), mental health (*P*=0.30), but the domain related to emotional limitations attained a statistically significant result (*P*=0.00031).

QoL was also correlated with compliance to a GFD assessed by the CDAT questionnaire and dosage of autoantibodies for those patients with CD. Scores were similar in both DMCD and CD groups (*P*=0.688). There was good correlation between the positivity of antibody anti-EMA and poor adherence to the GFD (*P*=0.0381, Fisher's exact test), but without repercussions on QoL. In short, it is possible to conclude that each disease has its own impact in QoL, and the association of both diseases did not result in an important worsening of it.

## Discussion

Our data show that nutritional status and BMI analysis were within the normal range, despite the fact that mean waist circumference was higher than reference values in 53% of participants in DMCD, DM and CD groups. In the same way, dual energy X-ray absorptiometry results did not show significant differences on visceral fat area or percentage of body fat among the groups evaluated, although we clearly observed an increase in visceral fat area in those in the CD group. Pitoco *et al.*^21^ evaluated 120 patients with very similar characteristics to individuals recruited for this study and observed greater severity of subclinical atherosclerosis in DMCD subgroup than in individuals with type 1 diabetes or CD alone. Therefore, they suggested that the association of these two autoimmune diseases (T1DM and CD) could accelerate atherosclerosis.

Dietary deficiencies are common at diagnosis and over active CD, especially of folate and vitamins B12 and B6, due to the loss of protein and brush border enzymes in the proximal portions of the intestine, even in the absence of gastrointestinal symptoms.^22^ Rarely are gluten-free products fortified with folic acid and vitamins, distinct from what happens with regular food, and this could be an explanation why approximately one-half of individuals with CD followed up for 10 years persisted with vitamin deficiencies, even with adequate gluten restriction.^23, 24^ We found folic acid deficiency in the celiac patients enrolled in this study (DMCD and CD groups). They had between 6 and 12 years of diagnosis with good adhesion to the GFD and steady metabolic control. In contrast, the detection of lower serum magnesium concentrations in the DMCD and DM groups appeared to be an isolated finding. The effect of the association of T1DM and CD on folic acid deficiency and other hydrosoluble vitamins, as well the mechanisms responsible for this deficiency, are unknown. Nonetheless, the involvement of dietary deficiencies or changes in absorption and increased urinary excretion in the face of sustained hyperglycemia may play a role in it.^25^ However, published data indicate a potential link between magnesium deficiency, and cardiovascular risk factors and atherosclerosis, since magnesium is a natural calcium channel blocker on vascular smooth muscle and myocardium.^26^ Thus, a periodic laboratory evaluation could be advisable to identify individuals, who are deficient and therefore most likely to benefit from supplementation of oligoelements and vitamins.

Neither T1DM nor CD by itself or in association influenced laboratorial parameters of bone metabolism or BMD. This finding can be explained by the characteristics of the population evaluated in this study such as: (1) young adults with normal weight; (2) majority of women in reproductive age; (3) similar intake of calcium and vitamin D; (4) regular glycemic control, thus without chronic urinary calcium excretion; and (5) celiac patients with good adhesion to GFD, which somehow contributed to the formation of adequate bone mass or did not impact it negatively.

The analysis of the SF-36 questionnaire identified that patients with DM1 achieved lower scores than the control group in general health and vitality areas, suggesting that ‘having diabetes' requires more attention to lifestyle habits and daily treatment, resulting in an increased perception of the disease. Probably the complexities involved in managing the treatment influenced the QoL of these individuals. In addition, the presence of diabetes-related complications was associated with lower scores on domain role limitations due to emotional problems. Likewise, in a cohort of patients with T1DM using multiple doses of insulin, van Dijk *et al.*^10^ found a decrease in scores on the assessment by the SF-36 questionnaire and EuroQol-VAS, interpreted as resulting from the complexity of treatment.

The WESDR (Wisconsin Epidemiologic Study of Diabetic Retinopathy) evaluated the QoL in individuals with T1DM 10 years after the first phase of the study and concluded that the development of complications, especially cardiovascular diseases, as well as changes in employment (retirement, occupying lower positions and unemployment) were associated with worsening of QoL.^27^ Interestingly, we observed the presence of complications did not affect domains of QoL potentially related to limitations of the physical functional capacity or pain, but they had a major impact on the field concerning emotional limitations. Moreover, we observed a higher prevalence of complications in DM than in DMCD group, in spite of the similar duration of the disease.

Data published by Bakker *et al.*^3^ pointed out a significant negative impact on QoL related to concerns inherent to diabetes and social fears of adults with both diseases. Nevertheless, we observed that individuals with both diseases (DMCD) tended to achieve higher QoL scores, which means better general health when compared with DM, CD or control subjects. These results also diverge from data published by Hallert *et al.*^28^ that showed that CD patients had more difficulty in living with the disease than those with diabetes or the general population. They referred to a feeling of ‘being excluded' from the social context due to the reduced availability of gluten-free foods. This difference could be related to either good adherence to both treatments or to the fact that patients with difficulties in adapting to treatment were more permissive to food transgressions, omissions in insulin application and less worried about glycemic control. Therewith, they had SF-36 scores closer to healthy controls, without the perception of being sick or influenced by the disease in social life.

The limitation of this study is related to the sample size. The inclusion of only 15 individuals in each group could have influenced the lack of statistical significance of waist circumference data, the compartmentalized analysis of the total body weight or even other parameters related to QoL. However, as the group was matched for the presence of each disease with healthy controls, the analysis of nutritional health and QoL could be considered representative for young adults, since there is a low prevalence of the association of both diseases in the general population. Likewise, other studies have been published with small sample size and the authors also reported the difficulty in recruiting patients with T1DM and CD.^29, 30, 31, 32, 33^

Recently a multicenter randomized controlled trial (CD-DIET)^34^ has been designed to clarify the impact of a GFD on clinically relevant outcomes as metabolic control, bone health, glycemic variability and QoL in children and adults with type 1 diabetes and asymptomatic CD, making clear the need for specific studies directed to this population. In this sense, the importance of this study was to point out that T1DM associated with CD was not related to worsening QoL, nor did it have a negative impact on glycemic control in a population of individuals matched for age and gender with others, who have just one of these diseases and share similar social environment. This information seems to be valuable to understand the health-disease process and to contribute to the re-evaluation of promotion, prevention, treatment and rehabilitation of the patient's health.

## Figures and Tables

**Table 1:** Demographic characteristics of the patients enrolled in the study according to groups

*Groups*	*DMCD*	*DM*	*CD*	*HC*	P*-value*
	(n=*15)*	(n=*15)*	(n=*15)*	(n=*15)*	
Age (years)	37.4±13.4	35.5±12.5	38 ±11.6	36.8±12.4	0.955
BMI (kg m^−2^)	23.2±3.0	24.0±3.1	22.9±3.6	23.1±2.7	0.794
Sex (M/F)	3/12	3/12	3/12	3/12	
Duration T1DM (years)	19.8±9.4	22.2±9.7			0.493*
Duration CD (years)	6.4±3.7		12.2±9.2		0.0015*

Abbreviations: BMI, body mass index; CD, celiac disease; DM, type 1 diabetes mellitus; DMCD, type 1 diabetes mellitus and celiac disease; HC, healthy control; M/F, male/female.

*P-value: ANOVA or Student's *t-*test.

Values are means±s.d.

**Table 2:** Body mass composition of the women patients enrolled in the study according to groups

*Groups*	*DMCD*	*DM*	*CD*	*HC*	P*-value*
	(n=*12)*	(n=*12)*	(n=*12)*	(n=*12)*	
BMI (kg m^−1^)	23.2±3.0	24.0±3.1	22.9±3.6	23.1±2.7	0.794
WC >80 cm (*n*)	8	9	8	6	
VF >100 cm^2^ (*n*)	4	3	6	4	

Abbreviations: BMI, body mass index; CD, celiac disease; DM, type 1 diabetes mellitus; DMCD, type 1 diabetes mellitus and celiac disease; HC, healthy control; VF, visceral fat area; WC, waist circumference; M/F, male/female.

*P*-value: analysis of variance.

Values are means±s.d.

**Table 3:** Laboratory measurements of vitamins, minerals, and serum and urinary laboratory biomarkers of bone formation and remodeling and bone mineral density

*Group*	*DMCD*	*DM*	*CD*	*HC*	P*-value*
	(n=*15)*	(n=*15)*	(n=*15)*	(n=*15)*	
Vitamin B12 (pg ml^−1^)	562.9±298	499.9±242	414.4±187	384.2±147	0.132
Folic acid (ng ml^−1^)	9.2±3.3	15.1±5.4	8.1±4.1	12.4± 4.1	**<0.001**
Iron (μg dl^−1^)	72.1±32.1	73.6±33.8	86.7±29.9	95.3±367	0.184
Copper(μg dl^−1^)	96.3±42.5	81.3±29.9	82.5±39.8	78.1±28.9	0.522
Zinc (μg dl^−1^)	57±18.7	55.1±17.4	53.3±13.4	51.7±6.3	0.821
Magnesium (mg dl^−1^)	1.9±0.2	1.9±0.2	2±0.2	2.1±0.21	**0.002**
Vitamin D (ng ml^−1^)	22.8±6.8	23.1±8.2	26.2±12.9	19.3±8.2	0.255
Vitamin A (ng ml^−1^)	18.2±11	15.7±8.6	17.8±8.3	15.5±6.2	0.726
S-tCa (mg dl^−1^)	9.4±0.4	9.6± (0,6)	9.3± (0.4)	9.3±(0.4)	0.135
S-iCa (mg dl^−1^)	5±0.2	4.9±0.3	4.8±0.2	4.9±0.2	0.089
Phosphorus (mg dl^−1^)	3.5±0.7	3.5±0.6	3.3±0.5	3.5±0.5	0.605
OH-vit D (ng ml^−1^)	22.8±6.8	23.1±8.3	26.2±12.9	19.3±8.2	0.255
PTH (pg ml^−1^)	50.7±21.6	39.7±18.3	46.7±24	46.4±21.7	0.571
CTX (ng ml^−1^)	0.6±0.5	0.4±0.2	0.5±0.3	0.5±0.3	0.590
P1NP (ng ml^−1^)	78.3±89.1	58.6±45.6	52.7±50.3	58.4±36.7	0.654
U-Ca/24 h (mg vol^−1^)	111.8±103	113.7±101.4	126.6±105.6	115.1±54.5	0.978
24-h UPE (mg vol^−1^)	691±296.9	545.9±188	511.3±274.7	432.9±205.6	0.052
Bone mass (g cm^−^^2^)	1.1±0.1	1.2±0.13	1.1±0.13	1.15±0.1	0.454

Abbreviations: 24-h UPE, 24-hour urine phosphorus excretion; CD, celiac disease; CTX, C-telopeptide of type I collagen; DM, type 1 diabetes mellitus; DMCD, type 1 diabetes mellitus and celiac disease; HC: healthy control; OH-vit D, OH-vitamin D; P1NP, type I collagen the amino (N)-terminal extension propeptides; PTH, Parathyroid hormone; S-iCa, serum ionized calcium; S-tCa: serum total calcium; U-Ca/24 h: 24-hour urinary calcium.

*P*-value: analysis of variance.

Values are means±s.d. Statistically significant differences are indicated in bold.

**Table 4:** Results of multiple comparisons by Bonferroni test for dosage of folic acid and magnesium among all four groups

*Groups compared*	*Mean difference*	*s.e.*	P*-value*	*CI (95%)*	
				*Lower*	*Upper*
*Folic acid*					
DMCD vs DM	−5.96	1.58	**0.002**	−10.29	−1.63
DMCD vs CD	1.13	1.58	>0.999	−3.20	5.46
DMCD vs GC	−3.17	1.58	0.302	−7.50	1.16
DM vs CD	7.09	1.58	**<0.001**	2.76	1.42
DM vs GC	2.79	1.58	0.499	−1.54	7.12
CD vs GC	−4.29	1.58	0.053	−8.62	0.04
					
*Magnesium*					
DMCD vs DM	0.02	0.06	>0.999	−0.15	0.19
DMCD vs CD	−0.11	0.06	0.421	−0.28	0.06
DMCD vs GC	−0.20	0.06	**0.010**	−0.37	−0.04
DM vs CD	−0.14	0.06	0.186	−0.31	0.03
DM vs GC	−0.23	0.06	**0.003**	−0.40	−0.06
CD vs GC	−0.09	0.06	0.886	−0.26	0.08

Abbreviations: BMI, body mass index; CD, celiac disease; CI, confidence interval; DM, type 1 diabetes mellitus; DMCD, type 1 diabetes mellitus and celiac disease; HC, healthy control.

*P*-value: Bonferroni test. Statistically significant differences are indicated in bold.

**Table 5:** Result of eight domains of health status questionnaire SF-36 by group

*Group*	*DMCD*	*DM*	*CD*	*HC*	P*-value*
Physical functioning	89±15	76±23	73±28.7	88.7±9	0.066
Physical role limitations	70±39.2	61.7±38.8	65±39.9	90±15.8	0.127
Bodily pain	72.5±28.1	62.1±27.4	62.1±27.6	71±20.2	0.554
General health perceptions	47.5±15.4	45.3±19.2	57.4±19.4	62± 9.3	**0.020**^a^
Energy/vitality	55±19.2	47±24.3	62.7±20.8	71.7±18	**0.014**^a^
Social functioning	79.2±23.5	62.5±24.5	64.2±30.2	792±20	0.117
Emotional role limitations	53.3±43.3	51.1±48.6	68.9±38.8	77.8±30	0.233
Mental health	61.9±23.8	54.9±22.6	68.3±20.4	74.7±20	0.090

Abbreviations: CD, celiac disease; DM, type 1 diabetes mellitus; DMCD, type 1 diabetes mellitus and celiac disease; HC, healthy control.

a statistically significant differences between DM and HC groups; *n*=15 patients per group.

*P*-value: analysis of variance test.

The value for each domain of the questionnaire of quality of life ranges from 0 (the worst state health) to 100 (the best state health). Statistically significant differences are indicated in bold.

**Table 6:** Relationship among A1c and domains of health status questionnaire SF-36 in T1DM patients

	*DMCD*			*DM*		
	*Correlation*	P*-value*	n	*Correlation*	P*-value*	n
Physical functioning	0.188	0.501	15	0.412	0.127	15
Physical role limitations	0.049	0.861	15	0.115	0.684	15
Bodily pain	-0.054	0.849	15	0.368	0.177	15
General health perceptions	0.091	0.748	15	0.423	0.116	15
Energy/vitality	0.197	0.482	15	0.518	**0.048**	15
Social functioning	0.216	0.440	15	0.062	0.827	15
Emotional role limitations	0.122	0.666	15	0.063	0.822	15
Mental health	0.239	0.391	15	0.238	0.393	15

Abbreviation: T1DM, type 1 diabetes mellitus.

*P*-value: Pearson product-moment correlation coefficient. Statistically significant differences are indicated in bold.
